# External validation and calibration of risk equations for prediction of diabetic kidney diseases among patients with type 2 diabetes in Taiwan

**DOI:** 10.1186/s12933-024-02443-4

**Published:** 2024-10-09

**Authors:** Hsuan-Yu Su, Thi Thuy Dung Nguyen, Wei-Hung Lin, Huang-Tz Ou, Shihchen Kuo

**Affiliations:** 1 Institute of Clinical Pharmacy and Pharmaceutical Sciences, College of Medicine, National Cheng Kung University, 1 University Road, Tainan, 701 Taiwan; 2 Institute of Clinical Medicine, College of Medicine, National Cheng Kung University, Tainan, Taiwan; 3 Division of Nephrology, Department of Internal Medicine, National Cheng Kung University Hospital, College of Medicine, National Cheng Kung University, Tainan, Taiwan; 4 Department of Pharmacy, College of Medicine, National Cheng Kung University, Tainan, Taiwan; 5 Division of Metabolism, Endocrinology and Diabetes, Department of Internal Medicine, University of Michigan Medical School, Ann Arbor, MI USA

## Abstract

**Background:**

Most existing risk equations for predicting/stratifying individual diabetic kidney disease (DKD) risks were developed using relatively dated data from selective and homogeneous trial populations comprising predominately Caucasian type 2 diabetes (T2D) patients. We seek to adapt risk equations for prediction of DKD progression (microalbuminuria, macroalbuminuria, and renal failure) using empiric data from a real-world population with T2D in Taiwan.

**Methods:**

Risk equations from three well-known simulation models: UKPDS-OM2, RECODe, and CHIME models, were adapted. Discrimination and calibration were determined using the area under the receiver operating characteristic curve (AUROC), a calibration plot (slope and intercept), and the Greenwood-Nam-D’Agostino (GND) test. Recalibration was performed for unsatisfactory calibration (*p*-value of GND test < 0.05) by adjusting the baseline hazards of risk equations to address risk variations among patients.

**Results:**

The RECODe equations for microalbuminuria and macroalbuminuria showed moderate discrimination (AUROC: 0.62 and 0.76) but underestimated the event risks (calibration slope > 1). The CHIME equation had the best discrimination for renal failure (AUROCs from CHIME, UKPDS-OM2 and RECODe: 0.77, 0.60 and 0.64, respectively). All three equations overestimated renal failure risk (calibration slope < 1). After rigorous updating, the calibration slope/intercept of the recalibrated RECODe for predicting microalbuminuria (0.87/0.0459) and macroalbuminuria (1.10/0.0004) risks as well as the recalibrated CHIME equation for predicting renal failure risk (0.95/-0.0014) were improved.

**Conclusions:**

Risk equations for prediction of DKD progression in real-world Taiwanese T2D patients were established, which can be incorporated into a multi-state simulation model to project and differentiate individual DKD risks for supporting timely interventions and health economic research.

**Supplementary Information:**

The online version contains supplementary material available at 10.1186/s12933-024-02443-4.

## Introduction

Diabetic kidney diseases (DKDs) affect approximately 40% of patients with type 2 diabetes (T2D) and are among the leading causes of end-stage renal disease [1]. DKDs are characterized by an estimated glomerular filtration rate (eGFR) of < 60 mL/min/1.73 m^2^ and/or a urine albumin-creatinine ratio (UACR) of ≥ 30 mg/g for 3 months [1]. Patients with DKDs are at high risk of morbidity and mortality and have compromised quality of life and a substantial economic burden [2, 3]. Given the numerous burdens attributable to DKDs, timely intervention for early-stage DKDs (e.g., microalbuminuria), which is often asymptomatic and thus late-diagnosed, is recommended by current treatment guidelines [4]. Early detection (e.g., screening albuminuria or eGFR levels), diagnosis (e.g., confirming cases with persistent albuminuria), and treatment of DKDs for patients with T2D are crucial to delay DKD progression.

Several risk equation-based prediction models for individual-level simulation are available to estimate the risks of DKD-related clinical events among T2D patients. They can be used to stratify patients’ risks according to their demographic and clinical characteristics to inform individualized treatment selections, determine the value of novel drugs in health economic evaluations, and optimize healthcare resource allocation [5]. Several drugs with renal-protective effects have recently been suggested for T2D patients with DKDs, including glucagon-like peptide-1 receptor agonists, sodium-glucose cotransporter 2 inhibitors, and finerenone [6]. However, the majority of existing risk equations were developed using relatively dated data (e.g., 1977 − 2009) from Caucasian populations in clinical trial settings [7, 8]. The risk equations developed using relatively dated data may not reflect modern practice, making it difficult to determine the value of recently developed treatment strategies for DKDs. The generalizability of clinical trial-derived risk equations to real-world populations with diverse clinical characteristics is not fully known. Moreover, the applicability of these risk equations to other races/ethnicities (e.g., Asians) should be carefully examined given the differences in the epidemiology and clinical presentation of DKDs across races/ethnicities [9].

Against this background, this study adapted well-known risk equations for prediction of DKDs (microalbuminuria, macroalbuminuria, and renal failure) using data from a real-world population with T2D in Taiwan.

## Methods

Overall, the present study comprised two analytic steps: (1) validating existing risk equations in 2/3 randomly selected study patients (validation set of study cohort) and then updating/recalibrating the risk equations if the model performance was not satisfied (i.e., inadequate discrimination or calibration), and (2) examining the performance of the updated/recalibrated risk equations from step 1 in the remaining 1/3 randomly selected study patients (test set of study cohort) (S1 Fig).

### Data source

A retrospective cohort was constructed using electronic health records (EHRs) from the National Cheng Kung University Hospital (NCKUH) in 2014 − 2021. NCKUH is a medical center in southern Taiwan. Details of its EHRs for health outcomes research are available elsewhere [10]. This study was approved by the Research Ethics Committees of NCKUH (A-ER-108097). The validation process of the risk equations followed the Transparent Reporting of a multivariable prediction model for Individual Prognosis Or Diagnosis (TRIPOD) checklist (S1 Table).

### Identification of risk equations for prediction of DKDs

We conducted a literature review to identify the risk equations for prediction of renal outcomes among patients with T2D. Only risk equations that comprise risk factors that are routinely monitored and collected in clinical practice were considered for adaptation in this study. The selection of risk equations was also based on the availability and transparency of risk equations, in terms of explicitly providing (1) the intercept/baseline hazard of the equation, (2) the coefficients of risk predictors in the equation, (3) the time framework for prediction, and (4) the operational definitions of risk predictors and renal outcomes of interest. The risk equations from the United Kingdom Prospective Diabetes Study Outcomes Model 2 (UKPDS-OM2) [8], Risk Equations for Complications Of type 2 Diabetes (RECODe) [7] and Chinese Hong Kong Integrated Modeling and Evaluation (CHIME) models were selected for the adaptation [11]. These risk equations are detailed in S2 Table.

### Study cohort for validating risk equations for prediction of DKDs

Taiwanese patients with at least two diagnosis records of T2D within 1 year between 2015 and 2017 were first identified from NCKUH’s EHRs, and the date of the first T2D diagnosis in 2015–2017 was set as the index date. Only patients having UACR < 30 mg/g and eGFR ≥ 30 mL/min/1.73 m^2^ in the year before the index date were included. An eGFR > 60 ml/min/1.73m^2^ is usually considered to rule out kidney disease, but patients having the eGFR between 30 and 60 mL/min/1.73m^2^ were not excluded in our study cohort because most glucose-lowering agents available nowadays remain recommended for patients with an eGFR of 30–60 mL/min.1.73m^2^. Hence, the inclusion of these patients would align with current treatment recommendations and preserve the generalizability of the study results to contemporary practice settings. Those aged less than 18 years at the index date, having renal failure records in the year before the index date, or without any UACR or eGFR data during the study follow-up period were excluded. Each study patient was followed up from the index date to loss to follow-up or the end of December 2021, whichever came first (S2 Fig).

Study subjects were 2:1 randomly separated into validation and test sets, where the validation set was used for validating and updating/recalibrating the selected risk equations from the RECODe, UKPDS-OM2, and CHIME models, and the test set was used for examining the performance of the updated/recalibrated risk equations.

### Measurements of risk predictors and renal outcomes

The risk predictors specified in the selected risk equations were measured in the year before or at the index date, including demographic and lifestyle characteristics (i.e., age, sex, ethnicity, smoking status), diabetes-related variables (i.e., diabetes duration, age at T2D diagnosis), medical history (i.e., heart failure, stroke, myocardial infarction, peripheral vascular disease, retinopathy, cataract, amputation, blindness), medication use (i.e., antihypertensive drugs, glucose-lowering agents excluding insulin, anticoagulants), physical examination (i.e., body mass index, systolic blood pressure, diastolic blood pressure), and laboratory data (i.e., UACR, eGFR, serum creatinine, glycated hemoglobin [HbA1c], total cholesterol, low-density lipoprotein, high-density lipoprotein, triglyceride, hemoglobin, white blood cell count) (S2 Table). Due to unavailability of diabetes duration in the EHRs, diabetes duration was assumed to be 5 years for all study subjects, as done in a previous study [12] based on similar Taiwanese T2D patient populations. The medical history and medication use were ascertained according to the International Classification of Diseases (ICD) diagnosis codes (S3 Table) and the Anatomical Therapeutic Chemical (ATC) Classification System (S4 Table), respectively. Missing data in these patient characteristics were handled using multiple imputation using a fully conditional specification with a discriminant function for categorical variables (i.e., smoking) and predictive mean matching for continuous variables (i.e., physical examination and laboratory data) [13]. 

The study outcomes for the risk prediction were microalbuminuria (defined as UACR 30–299 mg/g), macroalbuminuria (UACR ≥ 300 mg/g), and renal failure (eGFR < 15 mL/min/1.73 m^2^). These outcome events were ascertained as two consecutive measures with values defined above (i.e., UACR, eGFR) separated by at least 90 days. Of note, an eGFR value was estimated using the Modification of Diet in Renal Disease formula.

### Statistical analysis

The baseline characteristics of the study patients were analyzed using descriptive statistics, including the mean and standard deviation (SD) for continuous variables and frequencies and percentages (%) for categorical variables. The observed event risks were calculated as the number of events occurred during the study follow-up divided by the total number of study patients. The 7-year predicted probabilities for microalbuminuria and macroalbuminuria were estimated using risk equations from the RECODe model and that for renal failure was estimated using risk equations from the RECODe, UKPDS-OM2, and CHIME models.

The performance of the selected risk equations was evaluated in terms of discrimination and calibration. The discrimination was determined using the area under the receiver operating characteristic curve (AUROC), with the value ranging from 0.5 (indicating no discrimination) to 1 (indicating perfect discrimination) [14]. The calibration was assessed using the slope and intercept of the regression line plotted as the observed risks (*y* axis) versus predicted risks (*x* axis) of a given renal event of interest stratified by the decile of predicted risks. A slope of 1 and an intercept of 0 indicate ideal calibration. A calibration slope of above (below) 1 indicates that the risk equation underestimates (overestimates) the risk. In addition, the Greenwood-Nam-D’Agostino (GND) test was used to evaluate the agreement between the predicted and observed risks of renal events. A *p*-value of ≥ 0.05 suggests satisfactory calibration [15]. 

When unsatisfactory performance of the selected risk equations in validation set was revealed, as supported by inadequate discrimination (AUROC < 0.7) and unsatisfactory calibration (*p*-value of GND test < 0.05), an update/recalibration of the risk equations was conducted in this validation set of study cohort based on van Houwelingen et al.’s method [16] (S1 Method). Specifically, the recalibration was conducted by adjusting the baseline hazards of renal events through adding multipliers which addressed the variations in patients’ risks of developing renal events across different countries/healthcare contexts. Recalibration within each stratum (i.e., decile group) of predicted risks of renal events that accounted for the heterogeneity of individual risk profiles of developing renal events based on the miscalibration pattern was further performed if the unsatisfactory performance remained after the adjustment for the baseline hazards. Lastly, the performance of the updated/recalibrated risk equations was finally determined in test set of study cohort. As the sensitivity analyses, all of the above-mentioned analyses were repeated based on the restricted or refined definitions of renal outcomes (S5 Table) to test the robustness of the study results. The analyses were performed using SAS software version 9.4 and R software version 4.2.2. All significance tests were two-tailed and *p*-values < 0.05 were considered to indicate a statistically significant difference.

## Results

A total of 3,986 eligible patients were identified in the overall study cohort (S3 Fig), with an average age of 63.1 years, baseline UACR of 11.9 mg/g, and eGFR of 97.4 mL/min/1.73 m^2^ (Table 1). The study cohort was 2:1 randomly separated into 2,659 and 1,327 patients for validation and test sets, respectively, with comparable between-group patient characteristics except for age. Over a mean follow-up of 6.8 years, 35.7%, 6.0%, and 0.9% of patients developed microalbuminuria, macroalbuminuria, and renal failure, respectively (Table 2).

**Table 1:** Baseline characteristics of study patients (overall cohort and validation and test sets)

Characteristic	Overall cohort (*n* = 3,986)	Validation set (*n* = 2,659)	Test set (*n* = 1,327)	*p*-value*
*Demographic characteristics*				
Age, years (mean, SD)	63.08 (12.24)	62.81 (12.28)	63.63 (12.13)	0.0484
Female (%)	44.96%	44.60%	45.67%	0.5247
Smoking status (%)				
Current	4.24%	3.99%	4.75%	0.1119
Quit	2.86%	3.20%	2.19%	
Never	92.90%	92.82%	93.07%	
*Medical history (%)*				
Heart failure	3.79%	4.10%	3.17%	0.1454
Stroke	5.62%	5.38%	6.10%	0.3483
Myocardial infarction	1.93%	1.81%	2.19%	0.4112
Peripheral vascular disease	0.63%	0.75%	0.38%	0.1572
Retinopathy	3.54%	3.65%	3.32%	0.5926
Cataract	5.14%	5.11%	5.20%	0.9088
Amputation	0.05%	0.08%	0.00%	0.3176
Blindness	1.48%	1.50%	1.43%	0.8582
*Medication use (%)*				
Antihypertensive drugs	67.04%	66.19%	68.88%	0.0889
GLA (except insulin)	92.50%	92.89%	91.71%	0.1821
Anticoagulants	3.69%	3.84%	3.39%	0.4824
*Physical examination (mean, SD)*				
BMI, kg/m^2^	26.14 (3.13)	26.19 (3.14)	26.03 (3.09)	0.1230
SBP, mmHg	127.28 (6.70)	127.17 (6.58)	127.49 (6.93)	0.1571
DBP, mmHg	77.93 (5.21)	77.84 (5.22)	78.10 (5.18)	0.1400
*Laboratory data (mean*, SD)				
UACR, mg/g	11.93 (7.47)	11.82 (7.43)	12.15 (7.54)	0.1776
eGFR^†^, mL/min/1.73 m^2^	97.38 (33.67)	97.59 (33.47)	96.97 (34.06)	0.5837
Serum creatinine, mg/dL	0.80 (0.29)	0.80 (0.29)	0.80 (0.29)	0.8538
HbA1c, %	7.43 (1.40)	7.42 (1.38)	7.45 (1.43)	0.4358
Total cholesterol, mg/dL	165.56 (35.94)	165.82 (35.72)	165.05 (36.39)	0.5211
LDL cholesterol, mg/dL	101.19 (30.70)	101.40 (30.42)	100.77 (31.26)	0.5377
HDL cholesterol, mg/dL	51.05 (14.71)	51.01 (14.70)	51.11 (14.73)	0.8422
Triglyceride, mg/dL	135.37 (01.92)	134.10 (99.47)	138.23 (106.63)	0.2392
Hemoglobin, g/dL	13.09 (1.17)	13.10 (1.15)	13.07 (1.20)	0.4853
White blood cell count, ×10^9^/L	8.49 (1.64)	8.45 (1.60)	8.56 (1.73)	0.0558

Abbreviations: SD, standard deviation; GLA, glucose-lowering agent; BMI, body mass index; SBP, systolic blood pressure; DBP, diastolic blood pressure; UACR, urine albumin-to-creatinine ratio; eGFR, estimated glomerular filtration rate; LDL, low-density lipoprotein; HDL, high-density lipoprotein

*p-value < 0.05 represents a statistically significant difference in patient characteristics between validation and test sets

^†^eGFR was calculated based on the serum creatinine level using the Modification of Diet in Renal Disease (MDRD) study equation

**Table 2:** Number of events and event risk of renal outcomes (overall cohort and validation and test sets)

	Number of events	Follow-up (PYs)	Event rate (/100 PYs)	Event risk (%)
*Overall cohort (n = 3,986)*				
Microalbuminuria	1,421	19,614	7.24	35.65
Macroalbuminuria	238	26,131	0.91	5.97
Renal failure	36	26,997	0.13	0.90
*Validation set (n = 2,659)*				
Microalbuminuria	953	13,064	7.29	35.84
Macroalbuminuria	153	17,468	0.88	5.75
Renal failure	25	18,014	0.14	0.98
*Test set (n = 1,327)*				
Microalbuminuria	468	6,549	7.15	35.27
Macroalbuminuria	85	8,663	0.96	6.41
Renal failure	11	8,984	0.12	0.90

Abbreviations: PYs, person years

Table 3 summarizes the performance of the RECODe, UKPDS-OM2, and CHIME equations for renal outcomes in the validation set of patients. Discrimination using the RECODe equations was unsatisfactory for microalbuminuria (AUROC: 0.62) but acceptable for macroalbuminuria (0.76). Both the RECODe and UKPDS-OM2 equations yielded unsatisfactory discrimination for renal failure (0.64 and 0.60, respectively), but the CHIME equation provided acceptable discrimination (0.77) for renal failure. Furthermore, the risks of microalbuminuria and macroalbuminuria were underestimated (calibration slope > 1) using the RECODe equations and the risks of renal failure were overestimated (calibration slope < 1) using the RECODe, UKPDS-OM2, and CHIME equations (Table 3 and S4 Fig). Given these results for the validation set of patients, the RECODe equations for microalbuminuria and macroalbuminuria and the CHIME equation for renal failure were selected for further update and recalibration. The results of sensitivity analyses that utilized the modified definitions of renal outcomes were consistent with the primary findings (S5 Table).

**Table 3:** Performance of RECODe, UKPDS-OM2, and CHIME equations for renal outcomes in validation set of patients (before recalibration)

	RECODe		UKPDS-OM2		CHIME	
	Discrimination: AUROC	Calibration: slope/intercept (*p*-value of GND test)	Discrimination: AUROC	Calibration: slope/intercept (*p*-value of GND test)	Discrimination: AUROC	Calibration: slope/intercept (*p*-value of GND test)
Microalbuminuria	0.62	1.60/0.16 (< 0.001)	NA	NA	NA	NA
Macroalbuminuria	0.76	4.38/-0.05 (< 0.001)	NA	NA	NA	NA
Renal failure	0.64	0.33/-0.004 (< 0.001)	0.60	0.15/0/0063 (< 0.001)	0.77	0.16/-0.0025 (< 0.001)

Abbreviations: T2D, type 2 diabetes; RECODe, Risk Equations for Complications Of type 2 Diabetes; UKPDS-OM2, UK Prospective Diabetes Study Outcomes Model 2; CHIME, Chinese Hong Kong Integrated Modeling and Evaluation; AUROC, area under the receiver operating characteristic curve; GND: Greenwood-Nam-D’Agostino; NA, not applicable

Notes: (1) Acceptable discriminations are defined as AUROC values higher than or equal to 0.7; (2) A calibration slope of 1 and an intercept of 0 suggest ideal calibration; (3) A *p*-value < 0.05 indicates a significant difference between the predicted and observed event risks using the GND test, thereby implying unsatisfactory calibration

**Fig. 1:**
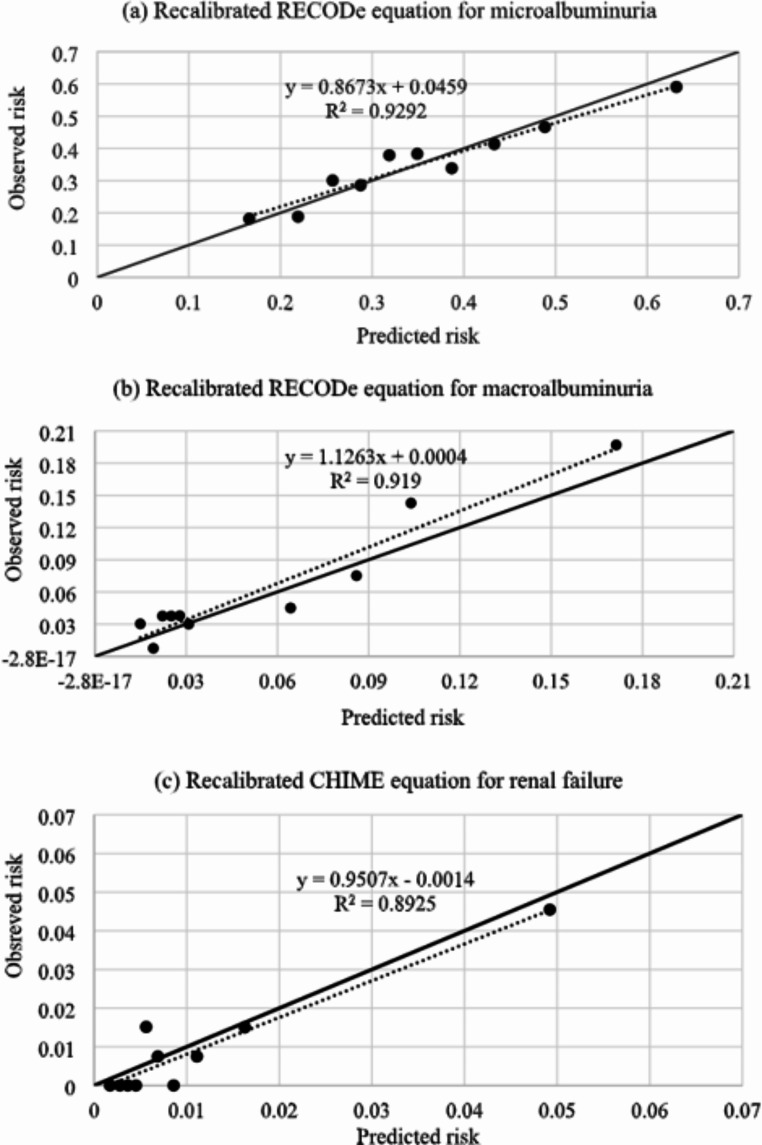
Calibration plots of the risk equations. **a** Microalbuminuria, **b** macroalbuminuria, and **c** renal failure among Taiwanese populations with T2D (after recalibration). Abbreviations: T2D, Type 2 diabetes; RECODe, Risk Equations for Complications Of type 2 Diabetes; CHIME, Chinese Hong Kong Integrated Modeling and Evaluation. Notes: (1) The recalibrated risk equations for (a) microalbuminuria, (b) macroalbuminuria, and (c) renal failure are shown below, where Risk_recalibrated_ represents the predicted risks after the recalibration, and Risk_RECODe−microalb_, Risk_RECODe−macroalb_ and Risk_CHIME_ represent the predicted risks of microalbuminuria, macroalbuminuria, and renal failure obtained using the original RECODe and CHIME equations, respectively: (a) Risk_recalibrated_ = 1 - exp (- exp (1.212 + ln (-ln (1 - Risk_RECODe−microalb_)))); (b) If Risk_RECODe−macroalb_ <2.4%, Risk_recalibrated_ = 1 - exp (- exp (0.343 + ln (-ln (1 - Risk_RECODe−macroalb_)))) If Risk_RECODe−macroalb_ ≥2.4%, Risk_recalibrated_ = 1 - exp (- exp (1.143 + ln (-ln (1 - Risk_RECODe−macroalb_)))); (c) Risk_recalibrated_ = 1 - exp (- exp (− 2.107 + ln (-ln (1 - Risk_CHIME_))))

After the recalibration procedures, the calibration slopes and intercepts for the RECODe equation for microalbuminuria, RECODe equation for macroalbuminuria, and CHIME equation for renal failure were improved (slope/intercept before recalibration: 1.60/0.1600, 4.38/-0.0500, and 0.16/-0.0025; slope/intercept after recalibration: 0.87/0.0459, 1.10/0.0004, and 0.95/-0.0014, respectively) (Tables 3 and 4; Fig. 1). All of the recalibrated equations demonstrated satisfactory calibration performance, i.e. calibration slope close to 1, intercept close to 0, and insignificant GND test result with a p-value > 0.05 (Table 4). The discrimination performances of these equations were similar before and after the recalibration.

**Table 4:** Performance of RECODe and CHIME equations for renal outcomes in test set of patients (after recalibration)

	Performance statistics	Microalbuminuria (RECODe)	Macroalbuminuria (RECODe)	Renal failure (CHIME)
Recalibration by overall baseline risks	Discrimination: AUROC	0.64	0.72	0.81
	Calibration: slope/intercept (*p*-value of GND test)	0.87/0.0459 (0.55)	1.78/-0.039 (0.06)	0.95/-0.0014 (0.46)
Recalibration by risk groups	Discrimination: AUROC	NA	0.72	NA
	Calibration: slope/intercept (*p*-value of GND test)		1.1/0.0004 (0.61)	

Abbreviations: T2D, type 2 diabetes; RECODe, Risk Equations for Complications Of type 2 Diabetes; CHIME, Chinese Hong Kong Integrated Modeling and Evaluation; AUROC, area under the receiver operating characteristic curve; GND: Greenwood-Nam-D’Agostino; NA, not applicable

Notes: (1) Acceptable discriminations are defined as AUROC values higher than or equal to 0.7; (2) A calibration slope of 1 and an intercept of 0 suggest ideal calibration; (3) A *p*-value < 0.05 indicates a significant difference between the predicted and observed event risks using the GND test, thereby implying unsatisfactory calibration

## Discussion

To our best knowledge, this study is the first to externally validate and recalibrate existing risk equations for predicting DKD progression in a Taiwanese population with T2D. Although the discrimination of the risk equations was acceptable, the equations underestimated the risks of microalbuminuria and macroalbuminuria and overestimated the risk of renal failure in this population. The risk equations’ performance in calibration was improved after rigorous recalibration procedures. These recalibrated risk equations can be incorporated into a multi-state simulation model to project and differentiate individual patient risks of DKD progression for supporting clinical management and economic research. This study also demonstrates an explicit, structured process for adapting and updating risk equations in different settings and populations.

### Comparative discriminative performance of risk equations for DKDs in the Taiwanese population

A discrepancy in model performance regarding discrimination between original development cohorts and external validation cohorts has been reported in previous studies [17]. Specifically, for albuminuria events, only the RECODe model differentiates microalbuminuria and macroalbuminuria events in two separate risk equations. Basu et al. validated the RECODe equation using a longitudinal cohort of African Americans and showed a relatively low discrimination on macroalbuminuria events in these patients compared to that observed in the original development cohort, which predominately comprised Caucasians (i.e., AUROCs: 0.77 versus 0.84) [17]. Similarly, a relatively low but acceptable discrimination of the RECODe equation was found for macroalbuminuria in our Taiwanese population (i.e., AUROC: 0.76). Moreover, the CHIME equation for renal failure yielded better discrimination (AUROC: 0.77) for the Taiwanese population compared to the 2 other equations for renal failure (0.64 and 0.60 from using RECODe and UKPDS-OM2, respectively) [7, 8]. 

These findings may be partly explained by the differences in patient clinical characteristics and time periods between the original development and external validation cohorts [18]. Specifically, the RECODe and UKPDS-OM2 models were built using data from selective and homogeneous Caucasian patients in clinical trial settings [7, 8] while the CHIME model was constructed using data from a real-world Asian patient cohort (similar to our study patients) [11]. The cohort used for the RECODe model comprised a high proportion of patients with established cardiovascular diseases or associated risk factors, including body mass index, HbA1c, and lipid profiles, which were much higher than those of our study population [7]. Moreover, both the RECODe and UKPDS-OM2 models were developed based on older data (i.e., 2001 − 2009 and 1977 − 1997, respectively) compared to data used for the CHIME model (i.e., 2006 − 2018) [7, 8, 11]. As medical technologies advance, older data might not reflect the modern clinical practice. For instance, the control of comorbid hypertension is considered important in current clinical management for patients with T2D [19], but most hypertensive cases in the UKPDS-OM2 model remained untreated given the few available treatment strategies and associated clinical recommendations at the time when the model was developed [8]. Therefore, owing to the similarity in the composition of the study cohort (i.e., Asians) and study period (i.e., modern era), the CHIME model showed the best discrimination for predicting the renal failure risk in the Taiwanese population (S6 Table).

### Recalibration of risk equations to improve predictive performance

The calibration results of the risk equations that indicate the accuracy of estimated risks for albuminuria and renal failure in Taiwanese patients were not satisfactory given a *p*-value < 0.05 of the GND test. Hence, existing risk equations should not be directly applied to Taiwanese patients without careful model updating and recalibration [20]. Specifically, the risk for albuminuria in Taiwanese patients was underestimated by the RECODe model, as supported by calibration slopes above 1 for both microalbuminuria (i.e., 1.6) and macroalbuminuria (i.e., 4.38). Such underestimation is expected given differences in the study cohort and associated underlying risks of albuminuria. That is, the RECODe model was developed using data from Caucasians primarily enrolled in clinical trials [7], who had a lower risk of albuminuria compared to that of Asians (e.g., Taiwanese) [21]. On the other hand, the risk for renal failure in Taiwanese patients was overestimated by all of the risk equations (i.e., calibration slope < 1), which might be due to the inconsistent definitions of renal failure and the underlying risks of renal failure across studies [22]. For example, renal failure in the CHIME model included dialysis and transplantation, which were ascertained according to associated diagnosis codes [11], whereas our study defined the presence of a renal failure event using two consecutive eGFR records of less than 15 mL/min/1.73 m^2^ separated by at least 90 days. The lower event rate of renal failure in our study compared to that of the CHIME model (0.13 versus 0.59 per 100 person-years) suggests the potential lower baseline risk of the event in our study cohort. The CHIME model was chosen for further recalibration given its best discriminative performance on renal failure.

To improve the accuracy of the estimated risks of albuminuria and renal failure for the RECODe and CHIME equations, efforts were made to optimize the predictive performance. Given the difference in renal event rates between the development cohort and our cohort, we first adjusted the baseline risk of the overall study cohort using a validated method (S1 Method) for all renal outcomes [14, 16]. However, the recalibrated result for macroalbuminuria remained undesirable following the initial adjustment at a cohort level, as supported by a miscalibration pattern observed in the calibration plot (S5 Fig). In particular, the underestimation was more extreme for high-risk patients (stratum 2) compared to that for low-risk patients (stratum 1), and thus the adjustment for the baseline hazards of the risk equations at a stratum level was further conducted. Our study demonstrated that recalibration of the risk equations improved predictive performances for renal outcomes, suggesting that updating and recalibration of the risk equations are needed to provide more precise estimations of event risks before these equations can be applied to target populations.

### Methodological efforts to ensure accuracy and applicability of risk equations

Several efforts were made to enhance the accuracy of estimated risks and applicability of the adapted risk equations for the Taiwanese population. First, sensitivity analyses, in which the definitions of renal outcomes were refined, were conducted. Consistent results across the primary and sensitivity analyses confirm the robustness of the study findings. Moreover, the risk equations were updated using validated methods to improve their prediction accuracy of renal outcomes. Second, a contemporary patient cohort based on EHRs was utilized to reflect a real-world setting with modern clinical practice, with easily obtained and measured clinical characteristics included as the predictors and risk factors. Lastly, we considered DKD progression from the status with normal albuminuria to three DKD subtypes, namely microalbuminuria, macroalbuminuria, and renal failure, which thoroughly depicted the disease progression. Albuminuria is an established risk factor for renal progression and is recognized as an early and sensitive marker for renal damage [23]. However, it is not typically considered in existing risk equations or simulation models.

The updated and recalibrated risk equations obtained in this study could be utilized in healthcare fields. For clinical practice, the risk equations can estimate a patient’s risk of developing a renal event conditional on their clinical characteristics to timely inform effective interventions. Several antidiabetic medications with renal-protective effects, such as sodium-glucose cotransporter 2 inhibitors, glucagon-like peptide-1 receptor agonists, and finerenone, are currently recommended for patients with DKDs [6]. Risk equation-based transition models, which comprehensively simulate the disease progression of DKDs, are useful for determining the specific renal effect of these medications regarding different DKD stages or progression and guiding personalized medicine. From a research perspective, this study offers a roadmap for model validation and recalibration. Notably, several risk prediction models for renal events are available today, but few have been externally validated and continuously updated, thereby restricting their generalizability to different settings and modern practice. Instead of developing a risk equation or model from scratch, adapting available risk equations and models to target populations or settings is generally suggested as an efficient approach [14, 24]. A risk equation with risk predictors that are transparently reported and routinely collected in clinical practice is usually preferred for further adaptation (e.g., external validation, updating, recalibration) [25]. 

### Limitations

First, several novel biomarkers (e.g., vascular adhesion protein-1, inflammatory chemokine CXCL12) [26, 27] that may be associated with renal progression were not considered in our risk equations. However, these biomarkers are not routinely measured in clinical settings and thus may have limited real-world applicability. Risk equations that contain risk factors or predictors that are routinely collected were thus selected in this study to enhance the applicability of our work to routine clinical practice. Second, the discrimination of the recalibrated RECODe risk equation for microalbuminuria remains unsatisfactory. A low discrimination of the RECODe model for microalbuminuria had been found in its original development cohort (AUROC: 0.62) [7]. Therefore, future research that will utilize more advanced methodologies or develop an updated risk equation is warranted to improve the predictive performance for microalbuminuria. It should be noted, however, that for a risk equation that aims to accurately predict risks of patients, its performance of discrimination might be less relevant than that of calibration [14]. Third, the information bias due to loss to follow-up, a common issue inherent to studies using EHRs, cannot be fully ruled out in this study. Several efforts were made to mitigate this concern, including restricting the study patients to those having at least two diagnoses of T2D for entry into the study cohort and at least one record of eGFR and UACR in both the baseline and follow-up period. Forth, the presence of a renal event of interest was not determined using a renal biopsy, since such a procedure is expensive and not routinely performed in clinical practice in Taiwan. Instead, we applied two consecutive laboratory records (e.g., eGFR less than 15 mL/min/1.73m^2^) separated by at least 90 days to confirm a renal event (e.g., microalbuminuria) in the primary analyses, and further conducted the sensitivity analyses which used the restricted or refined definitions of renal events (e.g., as the presence of at least one eGFR value less than 15 mL/min/1.73 m^2^ and at least one ICD diagnosis code; S5 Table). Consistent results between the primary and sensitivity analyses might have supported the robustness of our findings. Lastly, due to the unavailability of diabetes duration in NCKUH, we assumed a 5-year duration for all patients in this study, which might potentially diminish discriminative ability of our risk equations. However, the discriminative performance of our risk equations was consistent with that of the original risk equations, suggesting that such assumption might not affect the performance of the risk equations among Taiwanese T2D populations. Additionally, other risk predictors such as the history of diabetes-related complications, HbA1c data and use of glucose-lowering agents may have served as proxy indicators for diabetes duration in the risk equations.

## Conclusion

Through rigorous external validation and recalibration of existing risk equations, this study established updated risk equations for the prediction of DKD progression (microalbuminuria, macroalbuminuria, and renal failure) for real-world patients with T2D in Taiwan. These risk equations can be incorporated into a multi-state simulation model to project and differentiate individual patient risks of DKD progression for supporting clinical care and economic research. Future external validation is required before the application of our risk equations in other populations or healthcare settings.

.

## Electronic supplementary material

Below is the link to the electronic supplementary material.

Supplementary Material 1

## Data Availability

No datasets were generated or analysed during the current study.

## Abbreviations

DKDs: Diabetic kidney diseases
T2D: Type 2 diabetes
eGFR: Estimated glomerular filtration rate
UACR: Urine albumin-creatinine ratio
EHRs: Electronic health records
NCKUH: National Cheng Kung University
TRIPOD: Transparent Reporting of a multivariable prediction model for Individual Prognosis or Diagnosis
UKPDS-OM2: United Kingdom Prospective Diabetes Study Outcomes Model 2
RECODe: Risk Equations for Complications of type 2 Disbetes
CHIME: Chinese Hong Kong Integrated Modeling and Evaluation
HbA1c: Glycated hemoglobin
ICD: International Classification of Diseases
ATC: Anatomical Therapeutic Chemical
AUROC: Area under the receiver operating characteristic curve
GND: Greenwood-Nam-D’Agostino
