# Supplementary material for: External validation and calibration of risk equations for prediction of diabetic kidney diseases among patients with type 2 diabetes in Taiwan
Source: Cardiovasc Diabetol. 2024 Oct 9;23:357. doi: 10.1186/s12933-024-02443-4 (PMC11465834; doi:10.1186/s12933-024-02443-4)

**Supporting information**

**Supplementary Method 1. Recalibration method by van Houwelingen et al.**

The recalibration method adapted from van Houwelingen et al.’s study^1^ is specified as follows:

ln (- ln (1 - Observed_ncku_)) = *α_ncku_* + *β**ln (- ln (1 - Predicted_ncku_)), where:

Observed_ncku_: observed event risk of renal event,

Predicted_ncku_: predicted event risk of renal event obtained using original risk equations.

With *β* = 1, $\alpha_{ncku} =\ln\left( \frac{\ln\left( 1-Observed_{ncku} \right)}{\ln\left( 1-Predicted_{ncku} \right)} \right)$

The predicted event risk of renal event after recalibration (Risk_recalibrated_) is expressed as:

Risk_recalibrated_ = 1 - exp (- exp (*α_ncku_* + ln (-ln (1 - Predicted_ncku_))))

Reference:

1. Stat Med . 2000 Dec 30;19(24):3401-15.

**Supplementary Table 1. Transparent Reporting of a multivariable prediction model for Individual Prognosis Or Diagnosis (TRIPOD) checklist: prediction model validation.**

| **Section/Topic** | **Item** | **Checklist Item** | **Page** |
| --- | --- | --- | --- |
| **Title and abstract** | | | |
| Title | 1 | Identify the study as developing and/or validating a multivariable prediction model, the target population, and the outcome to be predicted. | 1 |
| Abstract | 2 | Provide a summary of objectives, study design, setting, participants, sample size, predictors, outcome, statistical analysis, results, and conclusions. | 3 |
| **Introduction** | | | |
| Background and objectives | 3a | Explain the medical context (including whether diagnostic or prognostic) and rationale for developing or validating the multivariable prediction model, including references to existing models. | 5 |
|  | 3b | Specify the objectives, including whether the study describes the development or validation of the model or both. | 5 |
| **Methods** | | | |
| Source of data | 4a | Describe the study design or source of data (e.g., randomized trial, cohort, or registry data), separately for the development and validation data sets, if applicable. | 6 |
|  | 4b | Specify the key study dates, including start of accrual; end of accrual; and, if applicable, end of follow-up. | 6 |
| Participants | 5a | Specify key elements of the study setting (e.g., primary care, secondary care, general population) including number and location of centres. | 6 |
|  | 5b | Describe eligibility criteria for participants. | 6 |
|  | 5c | Give details of treatments received, if relevant. | NA |
| Outcome | 6a | Clearly define the outcome that is predicted by the prediction model, including how and when assessed. | 7 |
|  | 6b | Report any actions to blind assessment of the outcome to be predicted. | NA |
| Predictors | 7a | Clearly define all predictors used in developing or validating the multivariable prediction model, including how and when they were measured. | 7 |
|  | 7b | Report any actions to blind assessment of predictors for the outcome and other predictors. | NA |
| Sample size | 8 | Explain how the study size was arrived at. | NA |
| Missing data | 9 | Describe how missing data were handled (e.g., complete-case analysis, single imputation, multiple imputation) with details of any imputation method. | 7 |
| Statistical analysis methods | 10c | For validation, describe how the predictions were calculated. | 8 |
|  | 10d | Specify all measures used to assess model performance and, if relevant, to compare multiple models. | 8 |
|  | 10e | Describe any model updating (e.g., recalibration) arising from the validation, if done. | 8,9 |
| Risk groups | 11 | Provide details on how risk groups were created, if done. | NA |
| Development vs. validation | 12 | For validation, identify any differences from the development data in setting, eligibility criteria, outcome, and predictors. | S6 Table |
| **Results** | | | |
| Participants | 13a | Describe the flow of participants through the study, including the number of participants with and without the outcome and, if applicable, a summary of the follow-up time. A diagram may be helpful. | S1 Fig |
|  | 13b | Describe the characteristics of the participants (basic demographics, clinical features, available predictors), including the number of participants with missing data for predictors and outcome. | Table 1 |
|  | 13c | For validation, show a comparison with the development data of the distribution of important variables (demographics, predictors and outcome). | S6 Table |
| Model performance | 16 | Report performance measures (with CIs) for the prediction model. | Table 3 |
| Model-updating | 17 | If done, report the results from any model updating (i.e., model specification, model performance). | Table 4 |
| **Discussion** | | | |
| Limitations | 18 | Discuss any limitations of the study (such as nonrepresentative sample, few events per predictor, missing data). | 22,23 |
| Interpretation | 19a | For validation, discuss the results with reference to performance in the development data, and any other validation data. | 19,20 |
|  | 19b | Give an overall interpretation of the results, considering objectives, limitations, results from similar studies, and other relevant evidence. | 19,20 |
| Implications | 20 | Discuss the potential clinical use of the model and implications for future research. | 22 |
| **Other information** | | | |
| Supplementary information | 21 | Provide information about the availability of supplementary resources, such as study protocol, Web calculator, and data sets. | NA |
| Funding | 22 | Give the source of funding and the role of the funders for the present study. | 26 |

**Supplementary Table 2. Detailed equations for renal events from RECODe, UKPDS-OM2, and CHIME models.**

| **Model** | **Renal outcome** | **Prediction time** | **Detailed risk equation** |
| --- | --- | --- | --- |
| RECODe^1^ | Microalbuminuria | 10 years | 1 - 0.78^exp(0.02114*age (years) + 0.16956 [if female] - 0.00804 [if Black] + 0.17342 [if Hispanic or Latino] + 0.28362 [if current smoker] + 0.00334*SBP (mmHg) + 0.22312 [if CVD history] + 0.28372 [if anti-hypertensive drug use] + 0.06584 [if GLA use] + 0.42199 [if anticoagulant use] + 0.13847*HbA1c (%) + 0.00034*TC (mg/dL) - 0.00970*HDL (mg/dL) + 0.67026*Scr (mg/dL) - 3.64) |
|  | Macroalbuminuria | 10 years | 1 - 0.94^exp(0.00733*age (years) + 0.27380 [if female] - 0.00556 [if Black] + 0.35630 [if Hispanic or Latino] + 0.10010 [if current smoker] - 0.00101*SBP (mmHg) + 0.25570 [if CVD history] + 0.24180 [if anti-hypertensive drug use] + 0.09015 [if GLA use] + 0.01091 [if anticoagulant use] + 0.09639*HbA1c (%) + 0.00009*TC (mg/dL) - 0.01135*HDL (mg/dL) + 1.14900*Scr (mg/dL) + 0.01335*UACR (mg/g) - 2.66) |
|  | Renal failure | 10 years | 1 - 0.97^exp(-0.01938*age (years) - 0.01129 [if female] + 0.08812 [if Black] + 0.23380 [if Hispanic or Latino] + 0.14830 [if current smoker] + 0.00303*SBP (mmHg) - 0.02164 [if CVD history] - 0.07952 [if anti-hypertensive drug use] - 0.12560 [if GLA use] + 0.03199 [if anticoagulant use] + 0.13690*HbA1c (%) - 0.00111*TC (mg/dL) + 0.00629*HDL (mg/dL) + 0.86090*Scr (mg/dL) + 0.00036*UACR (mg/g) - 0.23) |
| UKDPS-OM2^2^ | Renal failure | 1 year | 1 - exp(-exp(3.549 + 0.686 [if Afro-Caribbean] - 0.029*age at diagnosis of diabetes (years) - 0.869 [if female] - 0.054*BMI (kg/m^2^) - 1.031*eGFR (per 10 mL/min/1.73 m^2^) [if eGFR <60 mL/min/1.73 m^2^] - 0.487*eGFR (per 10 mL/min/1.73 m^2^) [if eGFR > 60 mL/min/1.73 m^2^] - 0.268*Hgb (g/dL) + 0.027*LDL (per 0.1 mmol/L) + 1.373 [if micro- or macro-albuminuria]+ 0.085*SBP (per 10 mmHg) + 0.029*WBC (per 10^9^/L) + 1.108 [if amputation history] + 0.732 [if blindness history])) |
| CHIME^3^ | Renal failure | 7 years | $\frac{1}{1+exp(\frac{log(7)-X\beta}{0.7981171})}$ where  Xβ = 6.166196 - 0.004849129*age (years) - 1.901472*10^-5^* (age - 45.86995)^3^ +  3.604495*10^-5^*(age - 61.54141)^3^ - 1.703024*10^-5^*(age - 79.03901)^3^ - 0.1409224*duration of diabetes (years) + 0.0007897111*(duration of diabetes - 0.04654346)^3^ - 0.00156559*( duration of diabetes - 4.960986)^3^ + 0.0007758793*(duration of diabetes - 9.963039)^3^ - 0.02053097*HbA1c (%)- 0.02178857*(HbA1c - 5.6)^3^ + 0.02582349*(HbA1c - 6.1)^3^ - 0.00403492*(HbA1c - 8.8)^3^ - 0.01317253*SBP (mmHg) - 5.375805*10^-6^*(SBP - 116)^3^ + 1.033809*10^-5^ *(SBP - 134)^3^ - 4.962282*10^-6^*(SBP - 153.5)^3^ + 0.02357291*DBP (mmHg) - 3.271212*10^-5^*(DBP - 64.66667)^3^ + 6.498808*10^-5^*(DBP - 77)^3^ - 3.227596*10^-5^*(DBP - 89.5)^3^ - 0.4526705*TRIG (mg/dL) + 0.08718189*(TRIG - 0.725)^3^ - 0.1285051*(TRIG - 1.299)^3^ + 0.04132321*(TRIG - 2.51)^3^ - 0.008416627*LDL (mg/dL) - 0.03346604*(LDL - 1.96452)^3^ + 0.06281429*(LDL - 2.9418)^3^ - 0.02934825*(LDL - 4.0562)^3^ - 0.154757*WBC (10^9^/L) + 0.004885142*(WBC - 5.03)^3^ - 0.008003012*(WBC - 7.2)^3^ + 0.00311787*(WBC - 10.6)^3^ + 0.5700462*Hgb (g/dL) - 0.00978487*(Hgb - 11.4)^3^ + 0.02168539*(Hgb - 13.65)^3^ - 0.01190052*(Hgb - 15.5)^3^ + 1.081168 [if female] - 0.2573695 [if diabetes] - 0.1490706 [if ex smoker] - 0.05581874 [if nonsmoker] - 0.2720151 [if anti-hypertensive drug use] - 0.1631902 [if cataract history] - 0.6716275 [if HF history]- 0.4899921 [if retinopathy history] - 0.3150657 [if PVD history] - 0.1800056 [if stroke history] |

Abbreviations: RECODe, Risk Equations for Complications Of type 2 Diabetes; SBP, systolic blood pressure; CVD, cardiovascular disease; GLA, glucose-lowering agent; TC, total cholesterol; HDL, high-density lipoprotein; Scr, serum creatinine; UACR, urine albumin creatinine ratio; UKPDS-OM2, UK Prospective Diabetes Study Outcomes Model 2; BMI, body mass index; eGFR, estimated glomerular filtration rate; Hgb, hemoglobin; LDL, low-density lipoprotein; WBC, white blood cell; CHIME, Chinese Hong Kong Integrated Modeling and Evaluation; DBP, diastolic blood pressure; TRIG, triglyceride; HF, heart failure; PVD, peripheral vascular disease.

Note:

The RECODe and UKDPS-OM2 consider the ethnicity of a patient as a risk factor (i.e., the binary variables for “black or not” [as 1 or 0 value] and “Hispanic/Latino or not” [1 or 0] in RECODe and “Afro-Caribbean or not” [1 or 0] in UKDPS-OM2). Since the present study targeted the race of Taiwanese population only, “0” value was applied for these binary variables. And the CHIME model does not include race/ethnicity in the risk equations, and in fact, the race of study participants for development of the CHIME model (i.e., Chinese patients in Hong Kong) would be similar with that in this study.

References:

1. Lancet Diabetes Endocrinol . 2017 Oct;5(10):788-798.
2. Diabetologia . 2013 Sep;56(9):1925-33.
3. PLoS Med. 2021 Jun 24;18(6):e1003692.

**Supplementary Table 3. Operational definitions of diseases of study interest using the International Classification of Diseases diagnosis codes.**

| **Disease** | **ICD-9-CM** | **ICD-10-CM** | **Data sources** |
| --- | --- | --- | --- |
| Cohort identification |  |  |  |
| Type 2 diabetes | 250.x0 or 250.x2, where x = 0−9 | E11 | OPD records |
| Medical history | | |  |
| Cardiovascular disease | | | ER, IPD and OPD records |
| Stroke | 430–434, 436 | I60, I61, I62, I63, I65, I66, I67.89 |  |
| Heart failure | 398.91, 402.01, 402.11, 402.91, 404.01, 404.03, 404.11, 404.13, 404.91, 404.93, 425.4, 425.9, 428 | I09.81, I11.0, I13.0, I13.2, I42.0, I42.2, I42.5, I42.7, I42.8, I42.9, I50 |  |
| Myocardial infarction | 410, 412 | I21, I22, I25.2 |  |
| Peripheral vascular disease | 250.7, 440–441, 443.1, 443.8–443.9 | E11.5, I70, I71, I77.819, I79.0, I73.1, I79.1, I79.8, I73.81, I73.89, I73.9 |  |
| Blindness | 368–369.9 | H54 |  |
| Retinopathy | 362, 250.50, 250.52 | E11.31, E11.32, E11.33, E11.34, E11.35, G45.3, H35, H34, H36 |  |
| Cataract | 366, 250.60, 250.62 | E11.4, H26, H25, E11.36, H28 |  |
| Amputation | 895–897, 997.6 | S78, S88, S98, T87.3, T87.4, T87.5, T87.8, T87.9 |  |
| Renal outcome |  |  |  |
| Renal failure | 403.01, 403.11, 403.91, 404.02; 404.03, 404.12, 404.13, 404.92, 404.93, 585.5, 585.6; 586, 588, V45.1, V56, V42.0 | I12.0, I13.11, I13.2, N18.5; N18.6, N19, N25, Z99.2, Z49, Z94.0 | ER, IPD and OPD records |

Abbreviations: ICD-9-CM, International Classification of Diseases, Ninth Revision, Clinical Modification; ICD-10-CM, International Classification of Diseases, Tenth Revision, Clinical Modification; OPD, outpatient department; ER, emergency room; IPD, inpatient department.

**Supplementary Table 4. Operational definitions of medications of study interest using Anatomical Therapeutic Chemical system.**

| **Medication** | **ATC code** | **Data sources** |
| --- | --- | --- |
| Antihypertensives | C02, C03, C04, C07, C08, C09 | OPD records |
| Glucose-lowering agents excluding insulin | A10B |  |
| Anticoagulants | B01AA, B01AB, B01AE, B01AF |  |

Abbreviations: ATC, Anatomical Therapeutic Chemical; OPD, outpatient department.

**Supplementary Table 5. Results of sensitivity analysis (with modified renal event definition).**

| **Renal event** | **Risk equation** | **Discrimination: AUROC** | **Calibration: slope/intercept (*p*-value of GND test)** |
| --- | --- | --- | --- |
| Microalbuminuria^*^ | RECODe | 0.63 | 1.82/0.33 (<0.001) |
| Macroalbuminuria^†^ | RECODe | 0.70 | 6.28/0.0284 (<0.001) |
| Renal failure (1)^‡^ | RECODe | 0.67 | 0.36/0.0055 (<0.001) |
|  | UKPDS-OM2 | 0.61 | 0.16/0.0058 (<0.001) |
|  | CHIME | 0.78 | 0.16/0.003 (<0.001) |
| Renal failure (2)^§^ | RECODe | 0.61 | 0.64/0.0028 (<0.001) |
|  | UKPDS-OM2 | 0.60 | 0.32/0.0162 (<0.001) |
|  | CHIME | 0.76 | 0.35/0.0038 (<0.001) |

Abbreviations: AUROC, area under the receiver operating characteristic curve; GND: Greenwood-Nam-D’Agostino; RECODe, Risk Equations for Complications Of type 2 Diabetes; UKPDS-OM2, UK Prospective Diabetes Study Outcomes Model 2; CHIME, Chinese Hong Kong Integrated Modeling and Evaluation.

^*^ Microalbuminuria was ascertained as the presence of at least one UACR value of 30-299 mg/g.

^†^ Macroalbuminuria was ascertained as the presence of at least one UACR value of ≥300 mg/g.

^‡^ Renal failure (1) was ascertain as having 1) two consecutive measures of eGFR <15 mL/min/1.73 m^2^ with a gap between two measures of ≥90 days and 2) at least one record of disease diagnosis for renal failure.

^§^ Renal failure (2) was ascertain as having 1) at least one eGFR value of <15 mL/min/1.73 m^2^ and 2) at least one record of disease diagnosis for renal failure.

Notes:

1. Acceptable discriminations are defined as AUROC values higher than or equal to 0.7.
2. A calibration slope of 1 and an intercept of 0 suggest ideal calibration.
3. A *p*-value <0.05 indicates a significant difference between the predicted and observed event risks using the GND test, thereby implying unsatisfactory calibration.

**Supplementary Table 6. Comparison of development cohorts for UKPDS-OM2, RECODe, and CHIME models and cohort in present study.**

|  | **NCKUH** | **CHIME** | **RECODe** | **UKPDS-OM2** |
| --- | --- | --- | --- | --- |
| **Region, year** | Taiwan,  2014−2021 | Hong Kong,  2006−2018 | United State/Canada,  2001−2009 | United Kingdom,  1977−1997 |
| **Data sources** | Real-world data  (NCKUH-EHRs) | Real-world data  (HK-CMS) | Trial data  (ACCORD trial) | Trial data  (UKPDS trial) |
| **Inclusion criteria** | 1. T2D 2. Over 18 years of age 3. eGFR ≥30 mL/min/1.73 m^2^ 4. Normo-albuminuria (UACR <30 mg/g) | 1. T2D 2. Over 20 years of age | 1. T2D 2. Aged 40-79 years 3. HbA1c ≥7.5% 4. Prior CVDs or risk factors for CVDs | - 1. Newly diagnosed T2D   2. Aged 25-65 years |
| **Ethnicity (%)** | Taiwanese (100%) | Chinese (92%),  Others (8%) | White (74%), Black (19%),  Hispanic/Latino (7%) | White (80%), Asian Indian (10%), Afro-Caribbean (10%) |
| **Baseline characteristics** | | | | |
| **Demographic characteristics** | | | | |
| Age, years, mean (SD) | 63.08 (12.24) | 60.00 (12.60) | 62.80 (6.70) | 53.30 (8.60) |
| Female (%) | 44.96% | 43.50% | 38.00% | 39.00% |
| Smoking status (%) | | | | |
| Current smoker | 4.24% | 13.70% | 12.00% | 31.00% |
| Past smoker | 2.86% | 18.80% | NA | 35.00% |
| Never | 92.90% | 67.50% | NA | 34.00% |
| **Medical history (%)** |  |  |  |  |
| Heart failure | 3.79% | 1.60% | NA^a^ | 0% |
| Cerebrovascular disease | 5.62% | 4.00% | NA^a^ | 0% |
| Peripheral vascular disease | 0.63% | 0.40% | NA | NA |
| Retinopathy | 3.54% | 0.80% | NA | 36.00% |
| Cataract | 5.14% | 4.30% | NA | NA |
| Amputation | 0.05% | 0.20% | NA | NA |
| Blindness | 1.48% | NA | NA | NA |
| **Medication use (%)** | | | | |
| Antihypertensive drugs | 67.04% | 35.20% | 84.00% | 12.00% |
| GLA (except insulin) | 92.50% | 18.20% | 83.00% | NA |
| Anticoagulants | 3.69% | NA | 3.00% | NA |
| **Physical examination, mean (SD)** | | | | |
| BMI, kg/m^2^ | 26.14 (3.13) | 25.60 (4.30) | 32.20 (5.40) | 27.5 (5.2) |
| SBP, mmHg | 127.28 (6.70) | 135.30 (15.40) | 136.50 (17.10) | 135.00 (20.00) |
| DBP, mmHg | 77.93 (5.21) | 77.60 (9.70) | 74.90 (10.70) | 82.00 (10.00) |
| **Laboratory data, mean (SD)** | | | | |
| UACR, mg/g | 11.93 (7.47) | NA | 99.20 (359.40) | NA |
| eGFR, mL/min/1.73 m^2^ | 97.38 (33.67) | 92.20 (28.40) | 90.90 (27.30) | NA |
| Serum creatinine, mg/dL | 0.80 (0.29) | NA | 0.90 (0.20) | 0.92 (NR) |
| HbA1c (%) | 7.43 (1.40) | 7.80 (1.70) | 8.30 (1.10) | 7.10 (1.50) |
| Total cholesterol, mg/dL | 165.56 (35.94) | NA | 183.20 (41.70) | 208.80 (42.50) |
| Triglyceride, mg/dL | 135.37 (101.92) | 141.70 (79.72) | 190.70 (145.80) | 208.20 (NA) |
| LDL cholesterol, mg/dL | 101.19 (30.70) | 116.00 (30.94) | 104.70 (33.80) | 135.50 (38.70) |
| HDL cholesterol, mg/dL | 51.05 (14.71) | 50.27 (11.60) | 41.80 (11.60) | 42.50 (7.70) |
| Hemoglobin, g/dL | 13.09 (1.17) | 13.70 (1.70) | NA | NA |
| WBC, ×10^9^/L | 8.49 (1.64) | 8.00 (2.30) | NA | NA |

Abbreviations: NCKUH, National Cheng Kung University Hospital; CHIME, Chinese Hong Kong Integrated Modeling and Evaluation; RECODe, Risk Equations for Complications Of type 2 Diabetes; UKPDS-OM2, UK Prospective Diabetes Study Outcomes Model 2; NCKUH-EHRs, National Cheng Kung University - Electronic Health Records; HK-CMS, Hong Kong Clinical Management System; ACCORD, Action to Control Cardiovascular Risk in Diabetes; UKPDS, United Kingdom prospective diabetes study; T2D, type 2 diabetes; CVDs, cardiovascular diseases; SD, standard deviation; NA, not available; GLA, glucose-lowering agent; BMI, body mass index; SBP, systolic blood pressure; DBP, diastolic blood pressure; UACR, urine albumin-to-creatinine ratio; eGFR, estimated glomerular filtration rate; LDL, low-density lipoprotein; HDL, high-density lipoprotein; WBC, white blood cell.

^a^ In cohort used for the development of the RECODe model, 36% of individuals had prior CVDs.

**Supplementary Fig 1. Overview of analytic steps**


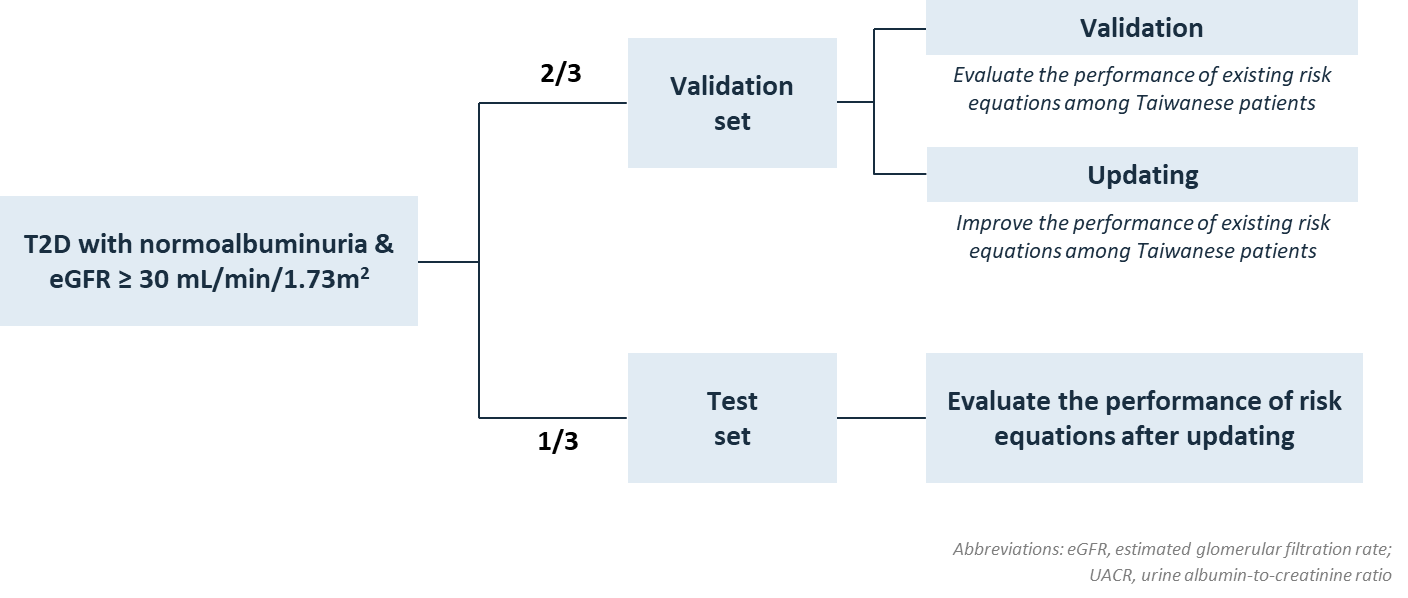


Abbreviations: T2D, type 2 diabetes; eGFR, estimated glomerular filtration rate.

**Supplementary Fig 2. Study scheme for identification of study subjects, risk predictors, and renal events.**


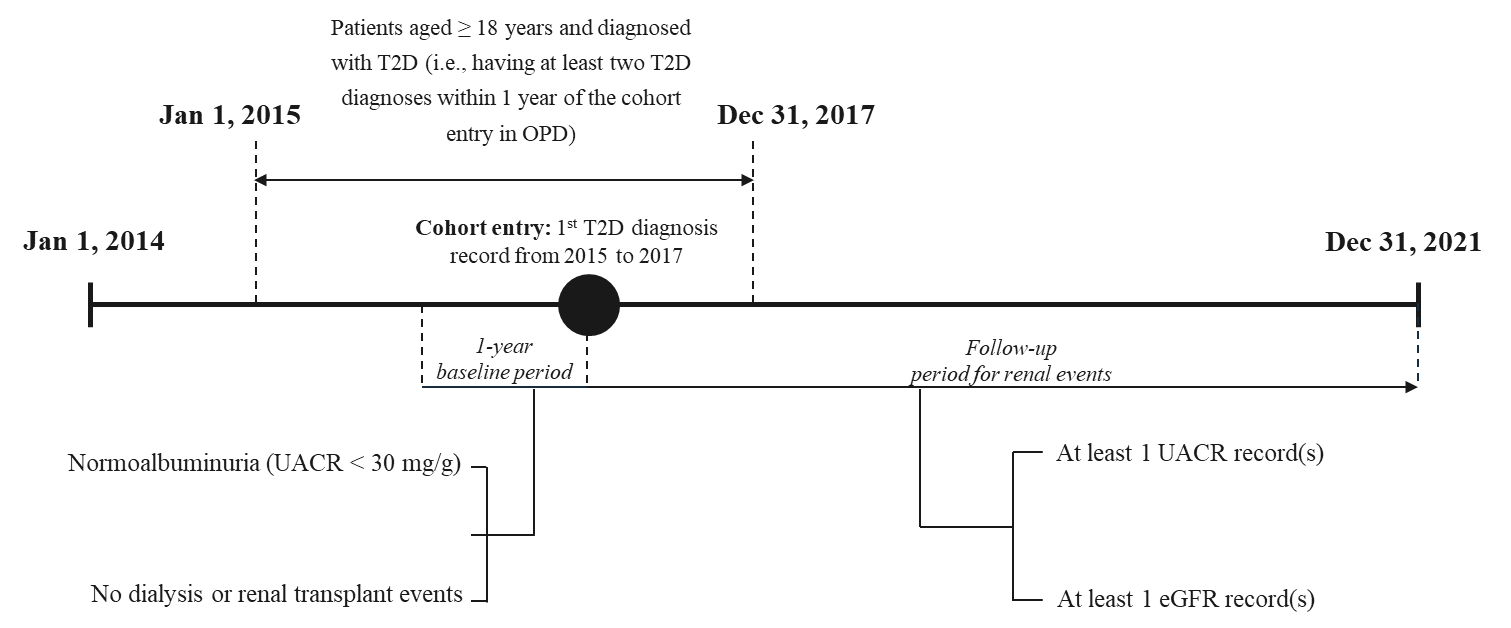


Abbreviations: OPD, outpatient department; T2D, type 2 diabetes; UACR, urine albumin-to-creatinine ratio; eGFR, estimated glomerular filtration rate.

**Supplementary Fig 3. Flowchart of cohort identification.**


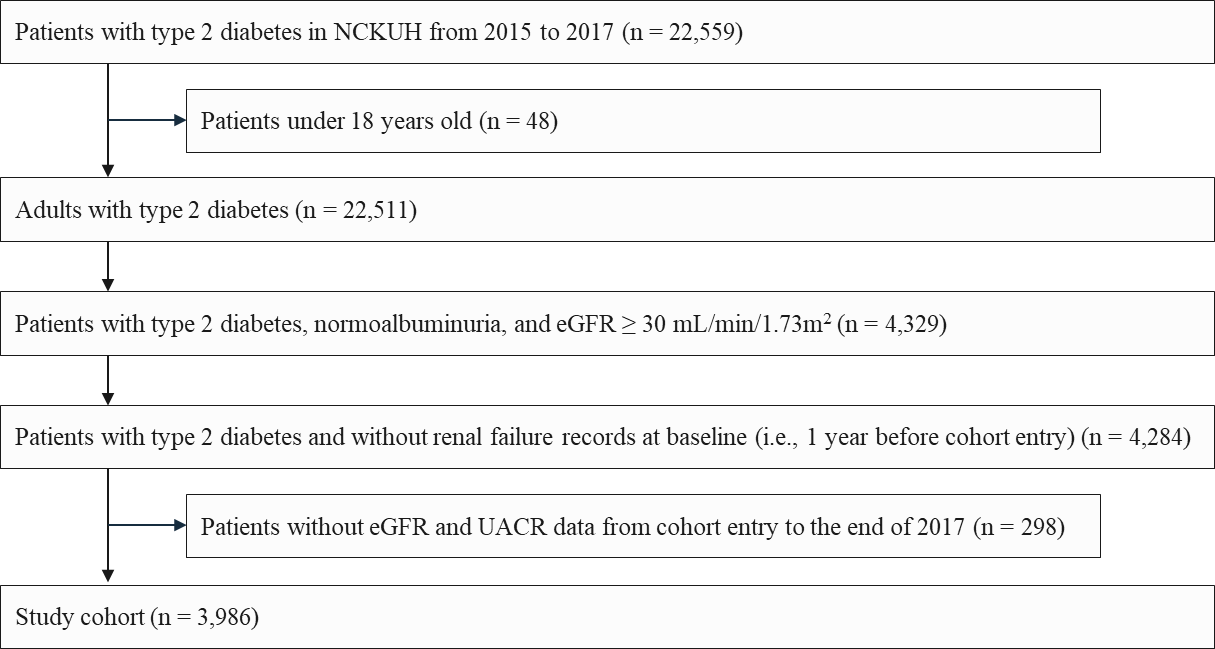


Abbreviations: NCKUH, National Cheng Kung University Hospital; eGFR, estimated glomerular filtration rate; UACR, urine albumin-to-creatinine ratio.

**Supplementary Fig 4. Calibration plots of the risk equations.**

(a) microalbuminuria, (b) macroalbuminuria, and (c) renal failure among Taiwanese populations with T2D (before recalibration).

Abbreviations: RECODe, Risk Equations for Complications Of type 2 Diabetes; UKPDS-OM2, UK Prospective Diabetes Study Outcomes Model 2; CHIME, Chinese Hong Kong Integrated Modeling and Evaluation.

**Supplementary Fig 5. Predicted and observed risks of macroalbuminuria stratified by decile of predicted risk.**


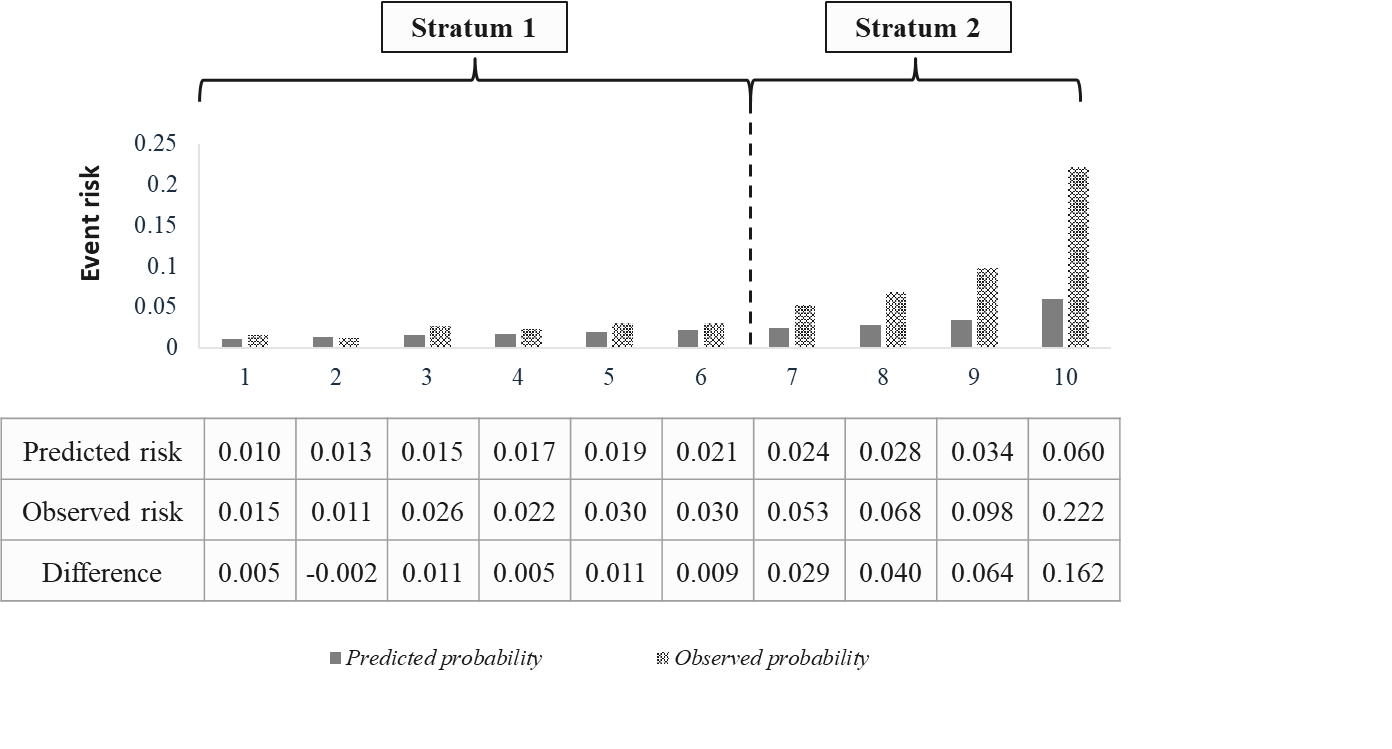

Supplement: Supplementary file 1 — Supplementary Material 1 [file 12933_2024_2443_MOESM1_ESM.docx]
